# Behavior and Musculoskeletal Effects of Chronic D-Galactose Treatment in Mice: Role of Heme Oxygenase-1

**DOI:** 10.3390/biom16040548

**Published:** 2026-04-08

**Authors:** Sally Wahba, Olufunto O. Badmus, Andrew R. Wasson, Elshymaa A. Abdel-Hakeem, Merhan Mamdouh Ragy, Hanaa Mohamad Ibrahim, Daniela Rüedi-Bettschen, David E. Stec

**Affiliations:** 1 Department of Physiology & Biophysics, Cardiovascular-Renal Research Center, Cardiorenal and Metabolic Diseases Research Center, University of Mississippi Medical Center, Jackson, MS 39216, USA; sally_wahba@mu.edu.eg (S.W.); obadmus@umc.edu (O.O.B.); ewk2ds@virginia.edu (A.R.W.); 2 Department of Medical Physiology, Faculty of Medicine, Minia University, Minya 61519, Egypt; shady@mu.edu.eg (E.A.A.-H.); myrehan.ragi@mu.edu.eg (M.M.R.); hanaa.mohamed@mu.edu.eg (H.M.I.); 3 Department of Psychiatry and Human Behavior, University of Mississippi Medical Center, Jackson, MS 39216, USA; drueedibettschen@umc.edu

**Keywords:** brain, oxidative stress, memory, aging

## Abstract

Chronic d-galactose (d-gal) treatment is a model to induce accelerated aging-like phenotypes in rodents. However, the sex differences in behavioral and musculoskeletal manifestations of this model are not well understood. Heme oxygenase-1 (HO-1) is a cytoprotective protein that may have anti-aging properties. The goal of this study was to better understand the sex differences in the behavioral and musculoskeletal effects of chronic d-gal treatment in C57BL/6J mice, as well as the role of HO-1 induction or inhibition. Eight-week-old male and female mice received daily saline or d-gal injections (500 mg/kg, s.c.) for 12 weeks. After this time, mice in the d-gal group were randomized into three groups (*n* = 6/group/sex): d-gal, d-gal + cobalt protoporphyrin (CoPP) (5 mg/kg, s.c. weekly), and d-gal + zinc deutroporphyrin bisglycol (ZnBG) (42 mg/kg, i.p. triweekly) for a period of 4 weeks. Open-field, novel-object recognition, Barnes maze, grip strength, micro-computed tomography (µ-CT), histology, and protein analysis were performed. Chronic d-gal treatment resulted in a sexual dimorphic response, with female mice being more prone to develop deficits in both short- and long-term spatial memory as well as in non-spatial memory. Male mice exhibited deficits only in long-term spatial memory when treated chronically with d-gal. Inhibition of HO-1 was protective in both females and males. Chronic d-gal treatment did not accelerate the development of osteoporosis or sarcopenia in either males or females. Our results demonstrate a sexual dimorphic response to the chronic effects of d-gal treatment on aging, with greater effects in females than in males, which is dependent on HO-1.

## 1. Introduction

Preventing or treating age-related diseases is a goal in modern medicine to reduce years of disability and the economic burden of aging. Dementia, senile osteoporosis, and sarcopenia are some of the most important causes of increased years of disability and loss of independence [1,2,3]. According to the Global Longevity Survey, the majority of responders from developed countries expressed their fear of having mental decline and Alzheimer’s disease (AD) as they age [4]. Additionally, the World Health Organization (WHO) reported that female deaths from AD and other dementias showed the highest rate of increment among other causes of death in the past two decades [5]. According to the Centers for Disease Control and Prevention (CDC) reports, senile osteoporosis, defined as a reduction in mean bone mineral density (BMD) in older adults compared with young adults of 2.5 or more standard deviations (SDs), is more common in aged women than men, with a prevalence of 19.6% in women compared to 4.4% in men [6]. Sarcopenia, defined as age-related loss of muscle mass and strength, is common in the elderly, with a general prevalence of 10–16% [7], and is more prevalent in women than in men across different patient cohorts [8,9]. These data underlie the importance of sex differences in the mechanisms and treatment options for these aging-associated conditions.

To study age-related diseases, accelerated-aging animal models were developed. D-galactose (d-gal)-induced aging is a widely used experimental model in rodents to simulate behavioral and musculoskeletal changes that occur with natural aging [10,11]. It was found to cause memory and locomotion impairment [12,13], bone loss [14], and skeletal muscle weakness and atrophy in rodents [15,16]. However, the sexual dimorphism in behavior and musculoskeletal phenotypes in C57BL/6 mice chronically treated with d-gal has not been well studied.

Previous studies have shown that nutraceuticals that can induce heme oxygenase-1 (HO-1) are protective against aging-related phenotypes in d-gal-treated mice [12,17]. Heme oxygenase-1 (HO-1) can be induced in essentially all cells of the body secondary to heme exposure, producing biliverdin, which is later converted to bilirubin, carbon monoxide (CO), and iron, which stimulates ferritin synthesis, all of which increase cells’ antioxidant capacity, promote their viability, regulate autophagy and apoptosis, and reduce inflammation [18,19]. Oxidative stress, ferroptosis, inflammation, increased senescence, and dysregulated autophagy are the proposed mechanisms for the development of aging-like phenotypes in the brains and musculoskeletal systems of mice chronically treated with d-gal. Previous studies have shown that nutraceuticals that can induce HO-1 are protective against aging-related phenotypes in d-gal-treated mice [12,17]. Still, the role of HO-1 induction and inhibition on the behavioral and musculoskeletal phenotypes in this model has not been previously studied. The goal of the present study was to investigate the effects of chronic d-gal treatment on spatial and non-spatial memory, as well as musculoskeletal phenotypes, in both male and female C57BL/6 mice, and to determine the impact of inducing or inhibiting HO-1 on these phenotypes.

## 2. Materials and Methods

### 2.1. Materials

The d-galactose (Thermo-Fisher Scientific, Waltham, MA, USA, cat #A12813.30) HO-1 inducer cobalt protoporphyrin (CoPP, Frontier Scientific, Logan, UT, USA #JH20-13893) and the HO-1 inhibitor zinc deutroporphyrin bisglycol (ZnBG, Frontier Scientific, Logan, UT, USA # JB16-10515) were prepared as previously described [20].

### 2.2. Animals

The experimental procedures and protocols of this study conformed to the National Institutes of Health (NIH) Guide for the Care and Use of Laboratory Animals. They were approved by the Institutional Animal Care and Use Committee of the University of Mississippi Medical Center (protocol #2024-1392). A total of 24 male and 24 female 8-week-old (24.8 ± 0.19 and 18.98 ± 0.24 g) C57BL/6 mice purchased from Jackson Labs (Bar Harbor, ME, USA) were tested in two phases. In experimental phase 1, animals were divided into 2 groups and treated daily with either subcutaneous (s.c.) saline injection (control, *n* = 6) or d-gal at a dose of 500 mg/kg BW for 12 weeks (*n* = 18) [21,22]. In experimental phase 2, which lasted for 4 weeks, the d-gal group was further subdivided into 3 equal subgroups (6 mice/group): the d-gal group continued as in phase 1, the d-gal + CoPP group was treated once weekly with CoPP at a dose of 5 mg/kg BW injected s.c. in addition to the daily d-gal treatment [23], and the d-gal + ZnBG group was treated 3 times/week with intraperitoneal (i.p.) injection of ZnBG at a dose of 42 mg/kg BW [24] in addition to the daily d-gal treatment. Animals were kept under a regular 12 h light/12 h dark cycle in a temperature- and humidity-controlled room and fed a normal chow diet with free access to food and water. After termination of the phase 2 experiments, mice were fasted for 10 h before tissues were harvested under deep isoflurane anesthesia, flash-frozen with liquid nitrogen, and stored at −80 °C until analysis.

### 2.3. Behavioral Tests

Behavioral tests were performed twice: at the end of phase 1 and at the end of the experiment. Before each test session, animals were acclimated to the testing room for 1 h to reduce potential confounds from stress associated with transport. All behavioral testing used EthoVision XT video tracking software Version 2.0 (Noldus, Wageningen, The Netherlands).

#### 2.3.1. Locomotion and Anxiety Analysis—Open Field Test (OF)

Mice were placed in the center of a square arena (40 × 40 cm) and were allowed to explore for 10 min. The arena was subdivided into 16 equal squares, with the middle 4 squares considered the center and the remaining squares the periphery. Velocity, total distance moved, and time spent in the center of the arena were measured [25].

#### 2.3.2. Recognitive Memory Analysis—Novel Object Recognition Test (NOR)

NOR was performed 24 h after the OF test in 2 sessions, with an inter-session interval of 30 min. In the 1st session, 2 identical objects were placed on opposite squares in the center of the OF arena. Mice were allowed to explore the 2 objects for 5 min. In the 2nd session, one of the objects was replaced with a new object (different shape and material), and mice were allowed to explore the familiar and the new objects for another 5 min. Primary (1ry) latency to the new object, defined as the time taken until the first visit to the object, total exploration time, discrimination index (DI), and preference index (PI) were measured. Indices and total exploration time were calculated as follows: total exploration time = time at the familiar object (FT) + time at the new object (NT); DI = (NT − FT)/(NT + FT); PI = NT/(NT + FT) [26,27].

#### 2.3.3. Spatial Memory Analysis—Barnes Maze (BM)

The Barnes maze (Stoelting Co, Wood Dale, IL, USA) consisted of a gray circular platform with a 100 cm diameter, elevated 100 cm above the ground. The platform had 20 regularly spaced 5 cm holes along the rim. One hole accommodated a 7 × 15 × 15 cm (w × l × d) escape box, whereas the other holes were equipped with a shallow sham escape box. Mice were first acclimated to the escape box for 2 min each. Then they were trained to locate the escape box using distinct visual cues on the walls surrounding the platform for 9 (for females) or 10 (for males) 3 min training sessions over 3 days (3 training sessions/day with an inter-trial interval (ITI) of 120 min). At 24 h after the last training session, the escape box was replaced with a sham escape box, and spatial memory was assessed with a 90 s 1st probe test (PT 1) [28,29]. At 48 h after the last training session, a 2nd 90 s probe test (PT 2) was performed to test long-term memory. Frequency, duration, primary (1ry) latency, and mean distance to the escape box were measured. The search strategy score (SSS) was calculated as previously described [30].

#### 2.3.4. Muscle Strength Analysis—Grip Test and Four-Limb Hanging Test

To measure grip strength, each mouse was held by its tail and allowed to grasp the grip grid (San Diego Instruments, San Diego, CA, USA) by its forelimbs only and then pulled steadily by its tail until release. Measurements were performed in triplicate (no ITI), and the average peak force was calculated over the 3 trials. After ~5 min of rest, mice were retested, allowing them to grasp the grid with all 4 limbs before peak force was assessed [31]. The 4-limb hanging test was performed as described previously, 2–4 h after the grip strength test [32]. Each mouse was placed in the middle of the grid, which was flipped over, and a stopwatch was used to count the seconds it remained hanging. If a mouse fell before one minute, with a maximum limit of 2 falls, it was picked up and placed again on the grid, and timing was resumed up to a maximum of 3 min of test duration.

### 2.4. In Vitro Cortical and Trabecular Bone Analysis

Isolated femurs dissected from mice at the time of euthanasia were preserved in 4% paraformaldehyde (PFA), thoroughly cleaned of attached soft tissues, wrapped in lightly wet gauze, and scanned with SKYSCAN 1276 µ-CT (BRUKER, Kontich, Belgium) as per the manufacturer’s instructions using an Al 0.5 mm filter and an X-ray setting of 60 KV and 200 µA. The highest camera power of 4096 × 4096 and pixel distance of 4 µm were used for maximum magnification. Standard phantom rods with a density of 0.3 and 1.25 g/cm^3^ were also scanned at the same settings, and their results were used to calibrate the software. Three-dimensional models were created with CT Vox software (Version 1), and bone mineral density (BMD) and tissue mineral density (TMD) for trabecular and cortical bones were measured with CTAn software (Version 1).

### 2.5. Immunohistochemistry

First, 5 µm paraffin-embedded female brain sections were deparaffinized and rehydrated, then immersed in citrate buffer at pH 6, and boiled for 45 min for antigen retrieval. After cooling, internal peroxidase (Thermo Scientific™ #350000) was added, followed by the blocking buffer (CytoVista™ Blocking Buffer, ThermoFisher Scientific, Waltham, MA, USA, #V11307). Slides were incubated overnight in the primary antibody solution of anti β-galactosidase (GLB-1 #ab203749) and diluting buffer (CytoVista™ Antibody Dilution Buffer, ThermoFisher Scientific, Waltham, MA, USA, # V11305) in a 1:500 ratio. Anti-rabbit secondary antibody was used at a 1:2000 dilution. A slide with only the secondary antibody served as a control. 3,3′-diaminobenzidine (DAB) staining was performed as per the manufacturer’s instructions (DAB Substrate Kit, AbCAM, Waltham, MA, USA, Peroxidase (HRP), with Nickel #SK4100). Briefly, the staining solution was prepared by mixing the buffer solution, DAB stock solution, hydrogen peroxide, and the nickel solution with 5 mL of distilled water. Next, 100 µL of the solution was added to each section and incubated for 10 min, then washed and imaged. A Mantra 2™ Quantitative Pathology Workstation (Quanterix, Billerica, MA, USA) at 10× magnification, and ImageJ (Version 1.54) were used to quantify the percentage of gray/black-stained areas.

### 2.6. Dihydroethidium (DHE) Staining

DHE staining was performed on 5 μm paraffin-embedded brain sections and quantified as previously described [20]. The DHE/DAPI ratio was calculated from images quantified using ImageJ [20].

### 2.7. Colorimetric β-Galactosidase Staining

Isolated male brains were preserved in 4% PFA for 7 days, then transferred to 10%, 20%, and 30% sucrose solutions over 3 days. Next, 30 µm-thick frozen sections were prepared for staining. The β-galactosidase staining solution was prepared according to the manufacturer’s instructions (Senescence β-Galactosidase Staining Kit, Cell Signaling Technology, Danvers, MA, USA, #9860). Brain sections were washed once with 1× PBS (200 µL/section), then 200 µL of 1× fixative solution was added to each section and incubated for 10–15 min at room temperature. The β-galactosidase staining solution (200 µL/section) was then added to the brain sections and then placed in an incubator at 37 °C for 8 h. Stained slides were imaged with a Mantra 2™ Quantitative Pathology Workstation, brightfield at ×10 magnification. Quantitative measurements of the % area of positive staining and the mean staining intensity were performed using ImageJ software.

### 2.8. Western Blot

Western blots were performed on homogenized livers or hindlimb muscles, and the total protein concentration in the lysate was measured. Aliquots of the lysate containing 30 µg protein/sample were used for Western blotting. Anti-CD36 (Cell Signaling Technology, Danvers, MA, USA, #14347) primary antibody was used at 1:1000 to assess fatty acid uptake. The mouse anti-HO-1 antibody (ENZO Life Sciences, Farmingdale, NY, USA, #ADI-OSA-110F) was used at a 1:2000 dilution. The mouse anti-HSP90 antibody (Santa Cruz Biotechnology, Dallas, TX, USA, #sc-13119) was used at a 1:1000 dilution, and the rabbit anti-GAPDH antibody (Proteintech, Rosemont, IL, USA, #10494-1-AP) was used at a 1:2000 dilution. Protein levels are expressed as relative amounts normalized to either HSP90 or GAPDH, as previously reported [33].

### 2.9. Statistical Analysis

Data were analyzed using GraphPad Software 10. Depending on the parameter assessed, a one-way ANOVA, a two-way/mixed-effect ANOVA, or a three-way ANOVA was used, followed by LSD post hoc tests for multiple comparisons as appropriate to compare treatment groups. A paired *t*-test was used to compare performance in the initial and final training sessions in the Barnes maze test. Results are presented as mean ± SEM, and a *p*-value ≤ 0.05 is considered statistically significant.

## 3. Results

### 3.1. Chronic D-Gal Treatment Does Not Induce HO-1 in the Liver

**Figure 1:**
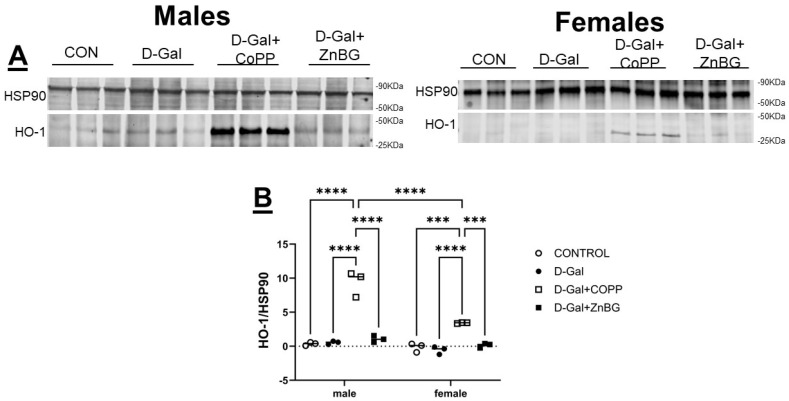
Chronic d-gal treatment does not induce HO-1 in the liver. (**A**) Representative Western blots. (**B**) Quantitation of the levels of HO-1 protein normalized to the levels of HSP90 protein and expressed as mean ± SEM. *n* = 3/group. HO-1: heme oxygenase-1, HSP90: heat shock protein 90, CON: control group, D-gal: d-galactose model group, *** *p* < 0.001, **** *p* < 0.0001. Statistical analyses were performed using a two-way ANOVA with an LSD post hoc test for multiple comparisons.

To determine the ability of d-galactose, CoPP, and ZnBG to induce HO-1, Western blot analysis of liver protein was performed at the end of the experimental protocol in both male and female mice. CoPP treatment resulted in significant induction of HO-1 in the livers of both male and female mice (F (3, 8) = 45.97, *p* < 0.001) as compared to control, d-gal-, and ZnBG-treated mice. Moreover, D-gal + CoPP-treated male mice had significantly higher liver HO-1 expression than their female counterparts (*p* < 0.0001) (Figure 1).

### 3.2. Female C57 Mice Are Less Anxious and More Explorative in New Environments than Male C57 Mice

Comparing male and female data after phase 1 of the experiment with a two-way ANOVA test showed significant sex differences between male and female mice in velocity, distance moved, frequency to the center, and time spent at the center of the OF arena. However, no significant differences between control and d-gal-treated mice in the OF test parameters were observed at this point. Comparing male and female data across the four experimental groups from the OF test after the end of phase 2 of the experiment revealed that female mice had significantly higher velocity (F (1, 39) = 21.47, *p* < 0.0001), longer distance moved (F (1, 39) = 21.40, *p* < 0.0001), and shorter latency to the center (F (1, 39) = 20.88, *p* < 0.0001) compared to male mice (Supplementary Figure S1).

**Figure 2:**
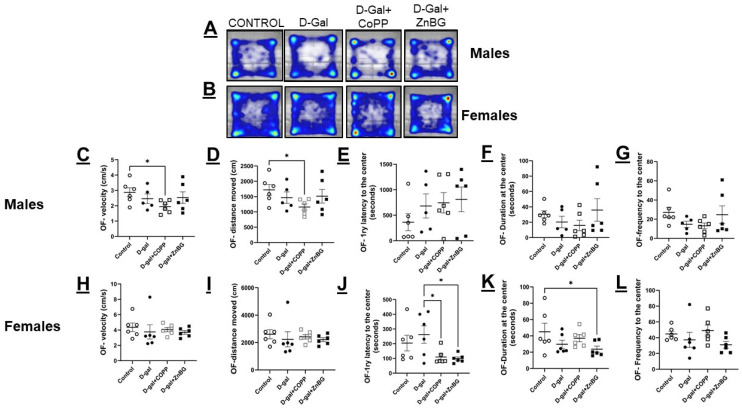
Assessment of locomotion and anxiety in male and female d-gal-treated mice. Heatmap of the movement in the open field arena of (**A**) male mice and (**B**) female mice. Inter-group quantitative analysis of the (**C**) velocity of male mice, (**D**) distance moved by male mice, (**E**) 1ry latency to the center in male mice, (**F**) frequency to the center in male mice, (**G**) duration at the center in male mice, (**H**) velocity of female mice, (**I**) distance moved by female mice, (**J**) 1ry latency to the center in female mice, (**K**) frequency to the center in female mice, and (**L**) duration at the center in female mice. Values are expressed as mean ± SEM, *n* = 6. CONTROL: control group, D-gal: d-galactose model group, * *p* < 0.05. Statistical analyses were performed using one-way ANOVA with an LSD post hoc test for multiple comparisons.

Comparing the four treatment groups in each sex separately, d-gal treatment alone did not decrease locomotion in male or female mice compared to the other groups (Figure 2A,B), but male mice treated with d-gal+ CoPP showed a significant reduction in their velocity and the distance moved in the open field test compared with control mice (*p* < 0.05), while those treated with d-gal + ZnBG showed no difference in locomotion compared to the control (Figure 2C,D). In females, there were no significant differences between the four groups in their locomotor activity (Figure 2H,I).

**Figure 3:**
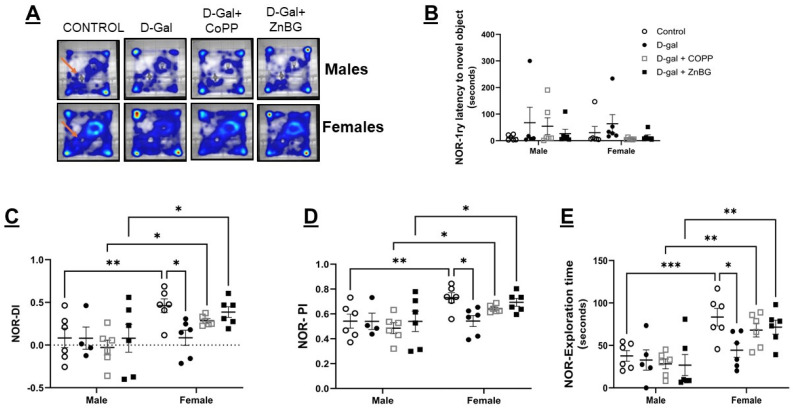
Assessment of recognitive memory with the NOR test in male and female d-gal-treated mice. (**A**) Heatmap of the movement in the NOR arena of male and female mice. Quantitative analysis of (**B**) 1ry latency to the novel object in male and female mice, (**C**) DI: discrimination index, (**D**) PI: preference index, and (**E**) total exploration time. Values are expressed as mean ± SEM, *n* = 6. CONTROL: control group, D-gal: d-galactose model group, * *p* < 0.05, ** *p* < 0.01, *** *p* < 0.001. Statistical analyses were performed using two-way ANOVA with an LSD post hoc test for multiple comparisons. Arrows indicate the location of the novel object.

One-way ANOVA for 1ry latency to the center was significantly different between treatment groups in female mice (F (3, 20) = 3.553, *p* < 0.05). Post hoc analysis showed a noticeable, albeit not significant, increase in the 1ry latency to the center of the open field arena in d-gal-treated mice. However, d-gal + CoPP-treated female mice showed a significant reduction in 1ry latency to the center as compared to d-gal-treated mice (post hoc, *p* < 0.05) (Figure 2E,J). D-gal + ZnBG-treated female mice also exhibited a significant reduction in 1ry latency to the center compared to d-gal-treated mice (*p* < 0.05). Still, contrary to the CoPP effect, this reduction was accompanied by a significant decrease in the duration spent at the center compared to the control (*p* < 0.05) (Figure 2J,K). No change in frequency or duration at the center was observed in male mice treated with d-gal alone or combined with CoPP or ZnBG (Figure 2F,G). Lastly, comparing male and female data across the four experimental groups from the NOR test after the end of phase 2 of the experiment showed that females had markedly longer exploration time (F (1, 39) = 30.13, *p* < 0.0001), higher DI (F (1, 38) = 12.38, *p* < 0.001), and PI (F (1, 38) = 12.38, *p* < 0.001) compared to males (Figure 3). An LSD post hoc test was performed for multiple comparisons, which showed that a sex difference was evident in the control and combined treatment with d-gal + CoPP and d-gal+ ZnBG groups but not in the model group treated only with d-gal where no significant difference was observed between male and female mice (*p* > 0.05) (Figure 3).

### 3.3. Chronic D-Gal Treatment Changes Learning Pattern and Magnitude in Both Male and Female Mice

**Figure 4:**
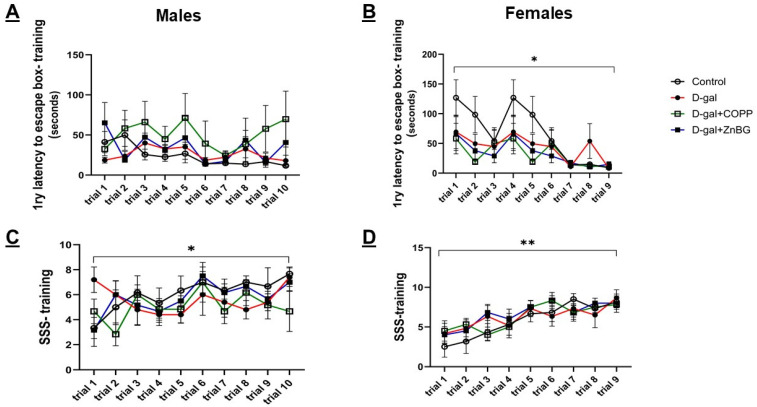
Assessment of learning in the training sessions of the Barnes maze (BM) test in male and female d-gal-treated mice. (**A**) Primary latency learning curves in male mice, (**B**) primary latency learning curves in female mice, (**C**) search strategy score (SSS) learning curves in male mice, and (**D**) search strategy score (SSS) learning curves in female mice. Values are expressed as mean ± SEM, *n* = 6. D-gal: d-galactose model group, significance is measured between the initial and final training session, * *p* < 0.05, ** *p* < 0.01. A paired *t*-test was used to compare performance in the initial and final training sessions.

To assess learning, 1ry latency to the escape box and the SSS curves during the BM training sessions were used. Similar to our findings with the previous tests, three-way ANOVA showed a highly significant sex dimorphism in favor of female mice (trial: F (8, 171) = 4.617, *p* < 0.001, sex: F (1, 171) = 24.02, *p* < 0.001; trial × sex: F (8, 171) = 2.656, *p* < 0.01; sex X treatment group; F (1, 171) = 4.039, *p* < 0.05). Next, the learning patterns of treatment groups within each sex were compared. Control male (Figure 4A,B) and control female mice (Figure 4C,D) exhibited the predicted learning curve, with an initial high primary latency and low SSS, followed by consistent progression toward the lowest latency and highest SSS. The magnitude of learning measured by comparing initial and final training session data with a paired *t*-test in control mice was statistically significant (male SSS (*p* < 0.05), male 1ry latency (*p* = 0.07), female SSS (*p* < 0.01), female 1ry latency (*p* < 0.05)). However, d-gal-treated male mice (Figure 4A,B) exhibited an opposite pattern, starting superior to controls and worsening progressively over the first half of the training sessions, then improving over the second half to return to their starting point. There was no significant change between initial and final data points. Female mice treated with d-gal (Figure 4C,D) also started superior to controls, but, similar to controls, showed progressive improvement. However, as in male mice treated with d-gal alone, there was no statistical significance between the initial and final data points. D-gal + ZnBG-treated male and female mice showed a trend toward improved SSS at the final session compared to the initial (*p* = 0.05, 0.07, respectively). D-gal + CoPP-treated female mice showed significant improvement in SSS at the final session compared to the initial (*p* < 0.05) (Figure 4).

### 3.4. Chronic D-Gal Treatment Impairs Recognitive Memory and Spatial Memory in Female C57 Mice

To assess recognitive (non-spatial) memory, the NOR test was performed and one-way ANOVA analysis was carried out to compare treatment groups/sex, which showed significant differences in female mice in total exploration time (F (3, 20) = 3.42, *p* < 0.05, DI (F (3, 20) = 5.634, *p* < 0.01), and PI (F (3, 2) = 5.634, *p* < 0.01. Female mice treated with d-gal showed a significant decrease in total exploration time (*p* < 0.01), DI (*p* < 0.001), and PI (*p* < 0.001) compared with control mice. ZnBG treatment with d-gal in female mice restored these parameters to control levels (*p* < 0.01) compared with the d-gal group. CoPP treatment with d-gal in female mice also slightly increased these parameters, but not significantly (Supplementary Figure S2E–H). On the other hand, there was no effect of chronic d-gal treatment in male mice (*p* > 0.05) (Supplementary Figure S2A–D).

**Figure 5:**
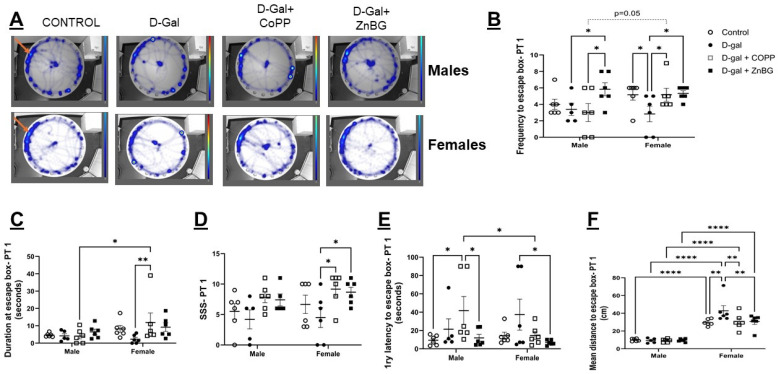
Assessment of spatial memory with 24 h (first) probe test of the BM test in male and female d-gal-treated mice. (**A**) Heatmap of movement in the BM arena of male and female mice, (**B**) quantitative analysis of the frequency to escape box area, (**C**) duration at the escape box area, (**D**) search strategy score (SSS), (**E**) 1ry latency to escape box area, and (**F**) mean distance to escape box area. Values are expressed as mean ± SEM, *n* = 6. CONTROL: control group, D-gal: d-galactose model group, PT1: first probe test, * *p* < 0.05, ** *p* < 0.01, **** *p* < 0.0001. Statistical analysis was performed using two-way ANOVA with an LSD post hoc test for multiple comparisons. Arrows indicate location of the escape box.

To evaluate spatial memory in males and females, the 24 and 48 h probe tests were used after 9–10 training sessions on the BM for female and male mice, respectively. In PT 1, after phase 1 of the experiment, the first latency to the target quadrant was significantly shorter in males than in females, but d-gal treatment did not have a significant effect on latency at this time point. After phase 2 of the experiment, the only sex difference detected was in mean distance to the escape box, which was higher in female mice as compared to males (F (1, 39) = 137.3, *p* < 0.0001) (Figure 5B,D,F). Post hoc analysis showed that d-gal-treated female mice had a significant reduction in the frequency of visits to the escape box location and a considerable increase in the mean distance to reach the escape box location compared to control mice (*p* < 0.05). D-gal + CoPP- and d-gal + ZnBG-treated groups showed a significant increase in the visit frequency and SSS, and a significant reduction in the mean distance compared to the d-gal-treated group (*p* < 0.05) (Figure 5C). CoPP treatment increased the duration spent at the former escape box location compared to d-gal-treated female mice (*p* < 0.01) (Figure 5C), while ZnBG treatment reduced the 1ry latency to the escape box location compared to control mice (*p* < 0.05) (Figure 5E). D-gal treatment in male mice did not cause any significant change compared to control mice (Figure 5B–F). However, CoPP-treated male mice exhibited a significant increase in the 1ry latency to the escape box location compared to control and d-gal + ZnBG-treated mice (*p* < 0.05) (Figure 5E). These results may reflect impairment in locomotion rather than in memory. D-gal + ZnBG-treated male mice had a significantly higher frequency to the escape box location compared to d-gal- and d-gal + CoPP-treated groups (*p* < 0.05) (Figure 5B).

In phase 2, sex differences in the frequency to escape box location (F (1, 39) = 5.967, *p* < 0.05) and duration spent at the escape box location (F (1, 39) = 7.338, *p* < 0.01) were higher in female mice compared to male mice, as revealed by two-way ANOVA. There were also significant inter-group differences in the frequency to escape box location (F (3, 39) = 7.247, *p* < 0.001), as well as significant interaction of sex X treatment groups in the frequency and mean distance to the escape box (F (3, 39) = 3.115, F (3, 39) = 3.339, respectively, *p* < 0.05). Post hoc analysis showed that female mice treated with d-gal had significantly reduced frequency (*p* < 0.05) and duration spent at the escape box location (*p* < 0.01) and markedly increased 1ry latency and mean distance to reach the escape box location (*p* < 0.01) as compared to controls (Supplementary Figure S3C–G). Combined treatment with d-gal + CoPP or d-gal + ZnBG significantly improved duration spent (*p* < 0.01) and 1ry latency at the escape box location (*p* < 0.05) compared to d-gal-treated female mice (Supplementary Figure S3D,F). The combined treatment of d-gal + ZnBG improved the mean distance to the escape box location compared to d-gal-treated female mice (*p* < 0.05) (Supplementary Figure S3G). D-gal-treated male mice showed a significant reduction in visit frequency to the escape box location and a significant increase in 1ry latency to the escape box location as compared to control mice (*p* < 0.05), which was reversed by ZnBG treatment (Supplementary Figure S3C,F).

### 3.5. ZnBG Treatment Reduces Senescence in the Female Hippocampus

**Figure 6:**
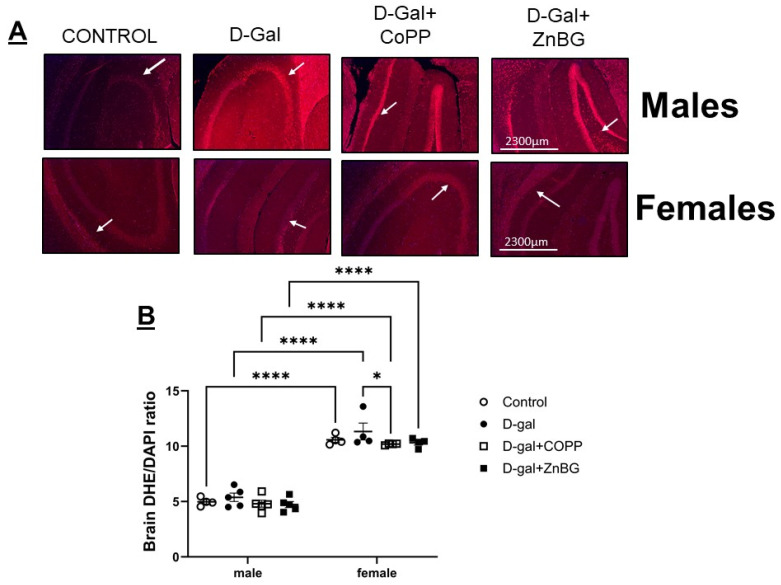
Assessment of oxidative stress from d-gal-treated mice. Representative images and quantitative analysis of (**A**) representative images of DHE staining of hippocampal brain sections of male and female mice, and (**B**) quantification of DHE staining in hippocampal brain sections of male and female mice. Values are expressed as mean ± SEM, *n* = magnified with x10 lens, field size 2300 µm. * *p* < 0.05; **** *p* < 0.0001. Statistical analyses were performed using two-way ANOVA with an LSD post hoc test for multiple comparisons. Arrows indicate areas of high DHE staining.

DHE staining in the hippocampus revealed significantly higher oxidative stress in female brains as compared to males based on two-way ANOVA (F (1, 27) = 492.3, *p* < 0.0001); however, there were no differences detected between the experimental groups (Figure 6). Chronic d-gal treatment in male and female mice did not result in a significant increase in senescence marked by either colorimetric β-galactosidase stain in males (Supplementary Figure S4) or IHC with GLB-1 in females (Supplementary Figure S5). D-gal + CoPP-treated male mice showed no significant difference in oxidative stress or senescence levels. In contrast, d-gal + CoPP-treated females trended toward decreased oxidative stress levels compared to d-gal treatment alone (Figure 6). D-gal + ZnBG-treated female mice also showed a significant reduction in senescence compared to the d-gal-treated group (post hoc, *p* < 0.05) (Supplementary Figure S5).

### 3.6. Chronic D-Gal Treatment Does Not Decrease Muscle Strength in C57 Mice

To assess muscle strength, forelimb grip, hindlimb grip, and four-limb hanging time were measured. No difference was observed between the d-gal and control groups, neither in males nor in females. Additionally, no effect was seen with either CoPP or ZnBG treatment in males or females (Supplementary Figure S6). However, there was a highly significant sex difference in forelimb strength in male mice as compared to females (*p* < 0.0001) when compared after both phases of the experiment.

### 3.7. Sexual Dimorphism in Bone Thickness and Mineralization

**Figure 7:**
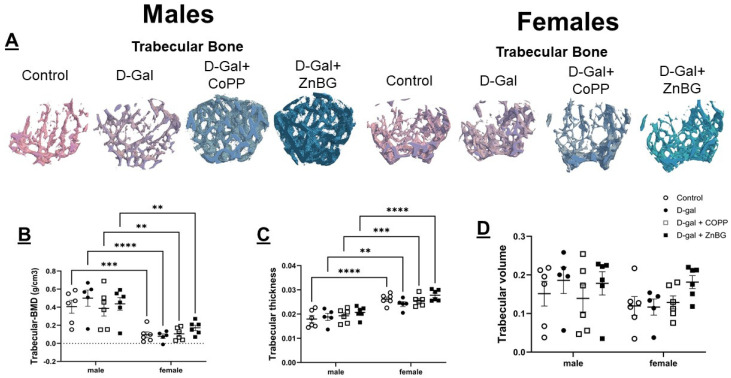
Assessment of trabecular bone density in d-gal-treated male and female mice. Representative 3D images of (**A**) trabecular bone in males and females, quantitative analysis of (**B**) trabecular bone BMD, (**C**) trabecular thickness, and (**D**) trabecular volume. Values are expressed as mean ± SEM, *n* = 6. D-gal: d-galactose model group, BMD: bone mineral density, ** *p* < 0.01, *** *p* < 0.001, **** *p* < 0.0001. Statistical analyses were performed using two-way ANOVA with an LSD post hoc test for multiple comparisons.

**Figure 8:**
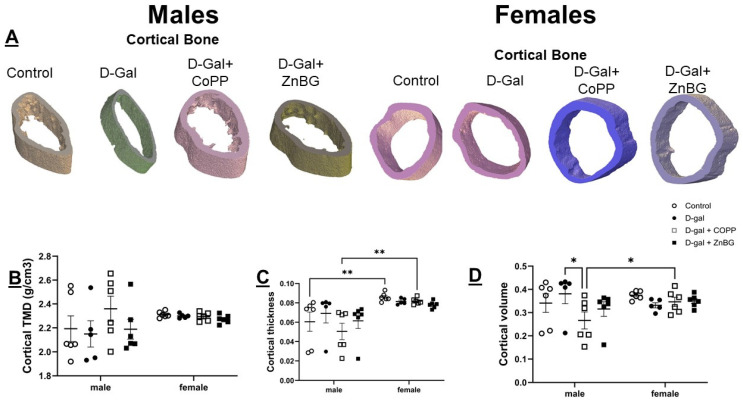
Assessment of cortical bone density in d-gal-treated male and female mice. Representative 3D images of (**A**) trabecular bone in male and female mice, quantitative analysis of (**B**) cortical bone TMD, (**C**) cortical thickness, and (**D**) cortical volume. Values are expressed as mean ± SEM, *n* = 6. D-gal: d-galactose model group, TMD: tissue mineral density, * *p* < 0.05, ** *p* < 0.01. Statistical analyses were performed using two-way ANOVA with an LSD post hoc test for multiple comparisons.

The assessment of trabecular and cortical bones by µ-CT showed significantly higher trabecular bone thickness based on two-way ANOVA (F (1, 38) = 72.45, *p* < 0.0001) but significantly lower trabecular bone mineral density (BMD) (F (1, 38) = 56.94, *p* < 0.0001) (Figure 7B,C) in female mice as compared to males. Cortical bone thickness was also significantly higher in females compared to males (F (1, 38) = 21.66, *p* < 0.0001) (Figure 8C). It was significantly lower in control and d-gal + CoPP-treated males as compared to females based on post hoc analysis (Figure 8C). Chronic d-gal-treated female mice demonstrated a trend toward reduction in cortical bone volume compared to the controls (Figure 8D). Chronic d-gal-treated male mice demonstrated higher cortical bone volume compared to d-gal + CoPP-treated mice (*p* < 0.05) but trended toward lower cortical tissue mineral density (TMD) (Figure 8B,D).

### 3.8. Chronic D-Gal Treatment Decreases Levels of CD36 in Female Mice

The assessment of CD36 protein levels in hindlimb muscle homogenate showed a significant reduction of CD36 in d-gal-treated female mice compared to the control group (*p* < 0.01), which was not improved with CoPP treatment but slightly increased with ZnBG treatment (*p* < 0.05) (Supplementary Figure S7). No significant differences in the protein levels of CD36 were observed in male mice treated with d-gal compared to controls (Supplementary Figure S7).

## 4. Discussion

This study was designed to examine sexual dimorphism in the behavioral and musculoskeletal effects of d-gal-induced aging in C57BL/6J mice. Additionally, we wanted to determine the role of HO-1 induction or inhibition in the behavioral and musculoskeletal effects of d-gal-induced aging. Our results show that CoPP injection significantly induced HO-1 protein levels in the livers of both male and female mice; however, d-gal treatment alone did not affect HO-1 levels in the livers of either male or female C57 mice in our study. This is different from what was previously reported, where chronic d-gal treatment reduced mRNA and the expression levels of HO-1 in the brain tissues of male C57 mice [12,17].

In the present study, there was a robust sexual dimorphism in the OF test, where female mice were much more mobile (higher speed and longer distance), and much less anxious (shorter latency and longer duration in the center) than male mice, which is similar to some previous reports [34,35] but different from the data reported by the Mouse Phenom Database (MPD) and other studies [36,37,38]. By analyzing data from the four experimental groups within each sex, there was no significant difference in locomotive activity or anxiety in either male or female C57 mice chronically treated with d-gal compared to control mice in the OF test. This finding helped us to interpret differences between d-gal-treated and control mice in BM and NOR tests as memory impairment rather than defective mobility. This finding was similar to the finding reported by Nam et al. [39] but different from the previous reports that found d-gal treatment to cause a reduction in the total distance moved in the OF test [12,40]. Notably, in these studies, either the mice were older at the start of the experiment than in this study, or the d-gal dose was twice that of ours. In this study, CoPP treatment in female mice chronically treated with d-gal had no effect on locomotion, but it reduced anxiety, as indicated by the shorter 1ry latency to the center. This is, in part, similar to what has been previously reported in female rats, where there was no change in either locomotion or anxiety after a single injection of CoPP [41]. Meanwhile, female mice treated with d-gal + ZnBG showed increased anxiety, as indicated by the shorter time spent in the center compared to the control group. On the other hand, CoPP treatment in male mice chronically treated with d-gal markedly reduced their locomotion, but it did not affect their anxiety levels. The observed reduction in locomotion is different from previous studies that showed chronic CoPP had no effect on locomotion in C57 male mice [23]. It has been shown that CoPP treatment can change the sex hormone profiles of male rats in the form of reduced testosterone and luteinizing hormone (LH) [42]. This change in testosterone levels in the early post-natal period can affect male brains and behavior [43,44]. However, the changes reported were in the opposite direction from the results shown here. This change might be due to the age at which testosterone deprivation occurred or the added effect of d-gal treatment, which was shown to reduce testosterone and elevate LH [45,46].

Testing sex differences in recognitive memory with the NOR test again showed female mice have higher exploratory behavior than male mice. Although previous studies reported a role of female sex hormones to improve brain networks and performance in memory related tests [47], other studies did not observe any significant difference in recognitive index (RI) between male and female C57 mice aged 2 months old, and a significant decrease in female RI at the age of 12 months old compared to age-matched males [48]. Surprisingly, by carrying out post hoc multiple comparisons tests, d-gal-treated female mice matched the male counterparts, suggesting that chronic d-gal treatment effects on female behavior could be in part due to changes in their sex hormonal profile. Studies using naturally aged C57 mice varied in their reported NOR results; some showed a decline in non-spatial memory in aged compared to young mice [48], while others reported no difference [49]. In our study, only female C57 mice chronically treated with d-gal showed impaired non-spatial memory, as indicated by the reduced exploration time, DI, and PI in the NOR test, while male C57 mice chronically treated with d-gal showed no difference in non-spatial memory compared to controls. Similar to our findings, Wei et al. reported reduced DI in female C57 mice chronically treated with d-gal but no change in exploration time [40], and Xiong et al. reported no difference in time spent at familiar and novel objects in male C57 mice chronically treated with d-gal [50]. However, others reported impaired DI in male C57 mice treated with d-gal [12,39,51,52]. Many studies on cognitive impairment models have shown HO-1 induction as a possible mechanism by which the proposed therapeutics exert their protective effect [53,54,55,56]. However, other studies have shown that HO-1 expression increased progressively with aging, and that patients with AD have over-expression of HO-1 in specific brain regions, one of which is the hippocampus, compared to non-demented subjects. This could explain why, in our study, the HO-1 inhibition with ZnBG in female mice had improved non-spatial memory, as indicated by the elevated exploration time, DI, and PI compared to the d-gal group.

In the present study, d-gal-treated C57 female mice had significant impairment in spatial memory assessed with the BM PT 1 and 2, in contrast to d-gal-treated C57 male mice that showed impairment in only long-term spatial memory (PT 2). These male data are similar to those previously reported with ICR male mice [57] but different from those reported in BALB/C male mice [58,59], all taking the same dose of d-gal treatment. Noticeably, previous studies using naturally aged (18-month-old) mice showed variable responses in the BM test, with some reporting significant differences between young and old mice [60] and some reporting the difference only being significant during the initial training and absent after training [61]. Previous studies using the d-gal model in C57 mice have preferably used the Morris Water maze test to assess spatial memory, which, in contrast to the BM test, imposes the extra stress from swimming on the tested animals [61], which might act as a second hit with the d-gal treatment leading to a more robust impairment in spatial memory in both male and female mice, even with lower doses of d-gal treatment [12,62,63,64]. D-gal + ZnBG-treated male and female mice showed significant improvement in their spatial memory compared to d-gal-treated mice, which could be secondary to the decrease in senescence in the hippocampus upon ZnBG treatment, as indicated by the reduced antigen–antibody complexes in IHC sections. Surprisingly, female mice treated with d-gal + CoPP also showed marked improvement compared to d-gal-treated females. We propose that this could be caused by CoPP inducing HO-1 in the vasculature, improving oxygenation and reducing ROS, as evidenced by the decreasing trend in the DHE signal. Previous studies have found that exercise increased HO-1 activity in the frontal and parietal cortex of aged rats, which attenuated cerebral amyloid angiopathy [65], and some studies using the d-gal model have found a reduction in HO-1 levels in the brain, and the restoration of its level was one of the mechanisms by which the proposed therapeutics exerted their protective effect [12,17]. On the other hand, ZnBG inhibiting HO-1 in the hippocampal glial cells could be reducing the free iron load, protecting them against ferroptosis and senescence, as previously reported in a myeloid cell knockout (KO) HO-1 mouse model or in a heterozygous HO-1 KO mouse model [66,67], and as evidenced by the markedly reduced senescence in this study. For future studies, we want to administer both the inducer and the inhibitor simultaneously, either systemically or directly inside the brain, to try to better understand the intricate role of HO-1 in the brain.

According to the MPD data, a significant reduction in grip strength was only observed between young and naturally aged male C57BL/6J mice, while no significant change was detected between young and aged female C57BL/6J mice. Additionally, young male mice had significantly stronger grip compared to young female mice, which was not observed when comparing aged animals [68]. In the current study, mice expressed a strong sex difference, similar to young mice in the MPD reports, but chronic d-gal treatment did not affect muscle power in either male or female C57 mice, despite the observed disturbance of muscle fatty acid metabolism in female C57 mice chronically treated with d-gal. These data are different from previous studies using the d-gal model, where they found significant attenuation of grip strength and grip time, and a decrease in gastrocnemius weight with changes in muscle fiber structure in d-gal-treated mice compared to controls [67,68]. CoPP and ZnBG treatment also had no effect on muscle power in this model. Previous studies have expressed the ability of HO-1 induction with CoPP to protect against skeletal muscle ischemia/reperfusion injury, and that ability depended upon the time of administration and was partly due to its ability to decrease leukocyte infiltration [69]; however, in a Duchan muscle dystrophy (DMD) mouse model, CoPP injection did not improve either exercise performance or the signs of inflammation and degeneration [70].

MPD data also show no significant difference in whole-body BMD between 6-month-old and 20-month-old C57BL/6J male or female mice [68]. Meanwhile, previous studies using d-gal-treated male mice (at a dose of 1 g/kg) have shown significant decrease in the distal femur BMD, cortical thickness, bone volume, and trabecular number, as well as a significant increase in trabecular separation compared to control animals [14] and a significant decrease in trabecular number and increase in trabecular separation in d-gal-treated SD male rats [71]. However, in the present study, d-gal-treated male mice had no change in their TMD, BMD, cortical, or trabecular volume compared to control mice, while d-gal-treated female mice had reduced bone volume and thickness, which was more prominent in the cortical bone than in the trabecular bone. Previous in vitro studies have shown that HO-1 induction with CoPP increases osteogenesis and differentiation of mesenchymal stem cells into osteoblasts [72], and HO-1 KO in vivo was associated with increased osteoclastic activity and decreased bone mass and BMD [73]. This is similar to our finding, where d-gal+ ZnBG treatment worsened female cortical TMD and thickness, but does not explain the improved trabecular BMD, thickness, and volume.

The current study had some limitations. (1) The inability of the d-gal model to induce aging-like phenotypes in the skeletal muscles of C57BL/6 J mice, hence the inability to describe the effect of HO-1 induction and inhibition. Older animals or a different strain may have been more suitable for studying the effects of HO-1 induction and inhibition on muscle aging. (2) The noticeably high individual variations (high SEM) in a small sample size (it was calculated with G-power based on previous studies’ findings) may have masked some of the behavioral effects of the model. (3) We were not able to measure the HO-1 expression level in the hippocampus of our mice. (4) We could not access beta galactosidase in the brains of males and females using the same experimental technique, which prevented us from performing inter-sex comparisons. (5) In our study design, we did not have a group treated with both CoPP and ZnBG to verify if the effects produced by either of them are abolished in the presence of the other, especially since we noticed similar effects for both in our results. (6) Finally, there was a noticeable sex difference that varied after d-gal treatment, but the effects of treatments on the level of sex hormones were not assessed in this study.

## 5. Conclusions

The results of this study show strong sex dimorphism in locomotion, anxiety, and exploration behavior in C57BL/6J mice, which was blunted after chronic d-gal treatment. This study also shows prominent sex dimorphism in the effect of chronic d-gal treatment on spatial and non-spatial memory, with females more sensitive than males to developing memory deficits after treatment. Based on the data from MPD and our results, C57BL/6J male mice appear to be resistant to developing senile osteoporosis, either by a natural aging process or chronic d-gal treatment, and C57BL/6J female mice appear to be resistant to developing senile sarcopenia, either by natural aging or chronic d-gal treatment. The d-gal model was not suitable in our experiment to recapitulate senile sarcopenia developed in aged male C57 mice. HO-1 plays an intricate role in the brain, where HO-1 inhibition was protective against non-spatial memory deficits, but for more complex spatial memory, both HO-1 induction and inhibition had a protective effect.

## Data Availability

Data will be made available upon a reasonable request.

## Supplementary Materials

The following supporting information can be downloaded at: https://www.mdpi.com/article/10.3390/biom16040548/s1, Figure S1: Comparison of males and females in the open field (OF) test; Figure S2: Inter-group comparison of males and females in the novel object recognition (NOR) test; Figure S3: Assessment of long-term spatial memory with 48 h (2nd) probe test of the BM test in male and female d-gal-treated mice; Figure S4: Assessment of hippocampal senescence in male mice; Figure S5: Assessment of hippocampal senescence in female mice; Figure S6: Assessment of grip strength in chronic d-gal-treated mice; Figure S7: Assessment of CD36 protein levels in the hindlimb muscles of d-gal-treated mice.

## Abbreviations

μ-CT	Micro-Computed Tomography
1ry	Primary
AD	Alzheimer’s Disease
BM	Barnes Maze
BMD	Bone Mineral Density
BW	Body Weight
CDC	Centers for Disease Control and Prevention
CO	Carbon Monoxide
CoPP	Cobalt Protoporphyrin
DAB	3,3′-Diaminobenzidine
D-gal	D-Galactose
DHE	Dihydroethidium
DI	Discrimination Index
DMD	Duchenne Muscular Dystrophy
FT	Time at Familiar Object
GLB-1	Anti-B-Galactosidase Antibody
GSS	Glutathione Synthase
HO-1	Heme Oxygenase-1
i.p.	Intraperitoneal
IHC	Immunohistochemistry
ITI	Inter-Trial Interval
KO	Knockout
LH	Luteinizing Hormone
MPD	Mouse Phenom Database
NOR	Novel Object Recognition
NT	Time at Novel Object
OF	Open Field
PFA	Paraformaldehyde
PI	Preference Index
PT 1	First Probe Test
PT 2	Second Probe Test
RI	Recognitive Index
s.c.	Subcutaneous
SSS	Search Strategy Score
TMD	Tissue Mineral Density
WHO	World Health Organization
ZnBG	Zinc Deutroporphyrin Bisglycol
