# Supplementary material for: Behavior and Musculoskeletal Effects of Chronic D-Galactose Treatment in Mice: Role of Heme Oxygenase-1
Source: Biomolecules. 2026 Apr 8;16(4):548. doi: 10.3390/biom16040548 (PMC13113933; doi:10.3390/biom16040548)
Supplement: Supplementary file 1 [file biomolecules-16-00548-s001.zip › biomolecules-4207034-supplementary.pdf]

Behavior and Musculoskeletal Effects of Chronic D-Galactose Treatment in Mice: Role of Heme Oxygenase-1

Sally Wahba, Olufunto O. Badmus, Andrew R. Wasson, Elshymaa A. Abdel-Hakeem,

Merhan Mamdouh Ragy, Hanaa Mohamad Ibrahim, Daniela Rüedi-Bettschen and David E. Stec

Supplementary Figures

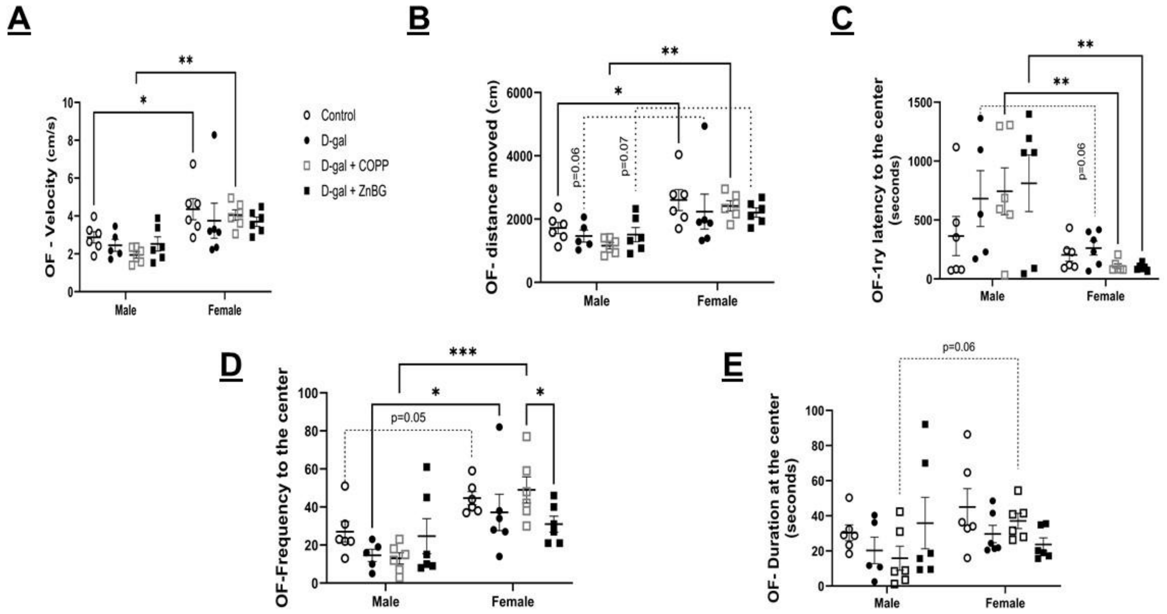

Figure S1. Comparison of males and females in the open field (OF)test. Quantitative difference between male and female in (**A**) velocity of movement (cm/s), (**B**) distance moved (cm), (**C**) primary (1ry) latency to center (s), (**D**) frequency to the center, and (**E**) duration at the center (s). Values are expressed as mean ± SEM, *n*= 6. D-gal; d-galactose model group, * P <0.05, ** P <0.01, *** P < 0.001. Statistical analyses were performed using a 2-way ANOVA with Tukey post hoc test.

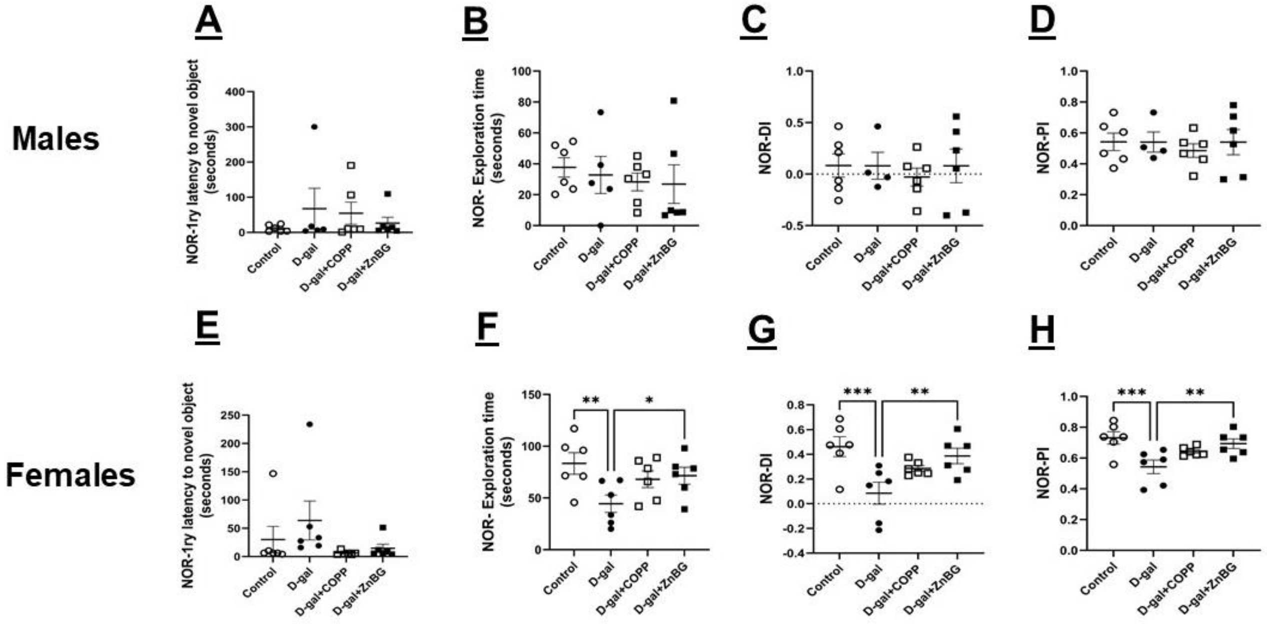

Figure S2. Inter-group comparison of males and females in the novel object recognition (NOR) test. Quantitative difference between male and female in (**A**) 1ry latency to novel object in males, (**B**) exploration time (s) in males, (**C**) discrimination index (DI) in males, (**D**) preference index (PI) in males, (**E**) 1ry latency to novel object in females, (**F**) exploration time (s) in females, (**G**) discrimination index (DI) in females, (**H**) preference index (PI) in females. Values are expressed as mean ± SEM, *n*= 6. CON; control group, D-gal; d-galactose model group, * $P < 0.05$, ** $P < 0.01$, *** $P < 0.001$. Statistical analyses were performed using a 2-way ANOVA with Tukey post hoc test.

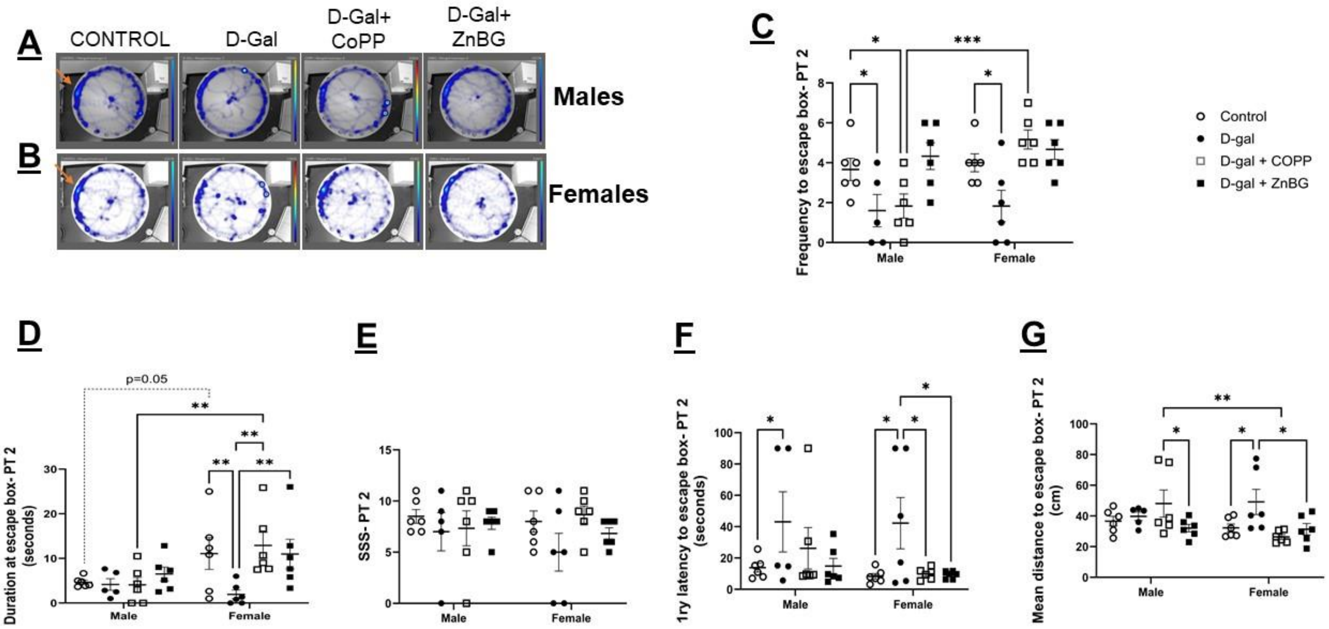

Figure S3. Assessment of long-term spatial memory with 48 h (2nd) probe test of the BM test in male and female d-gal-treated mice. Heatmap of the movement in the BM arena of (**A**) male mice and (**B**) female mice. Inter-group quantitative analysis of (**C**) frequency to escape box area in male and female mice, (**D**) duration at the escape box area in male and female mice, (**E**) SSS in male and female mice, (**F**) 1ry latency to escape box area in male and female mice, (**G**) mean distance to escape box area in male and female mice. Values are expressed as mean ± SEM, *n*= 6. CON; control group, D-gal; d-galactose model group, * P <0.05, ** P <0.01, *** P < 0.001. Statistical analyses were performed using a one-way ANOVA with LSD post hoc test for multiple comparisons. Arrows indicate location of the escape box.

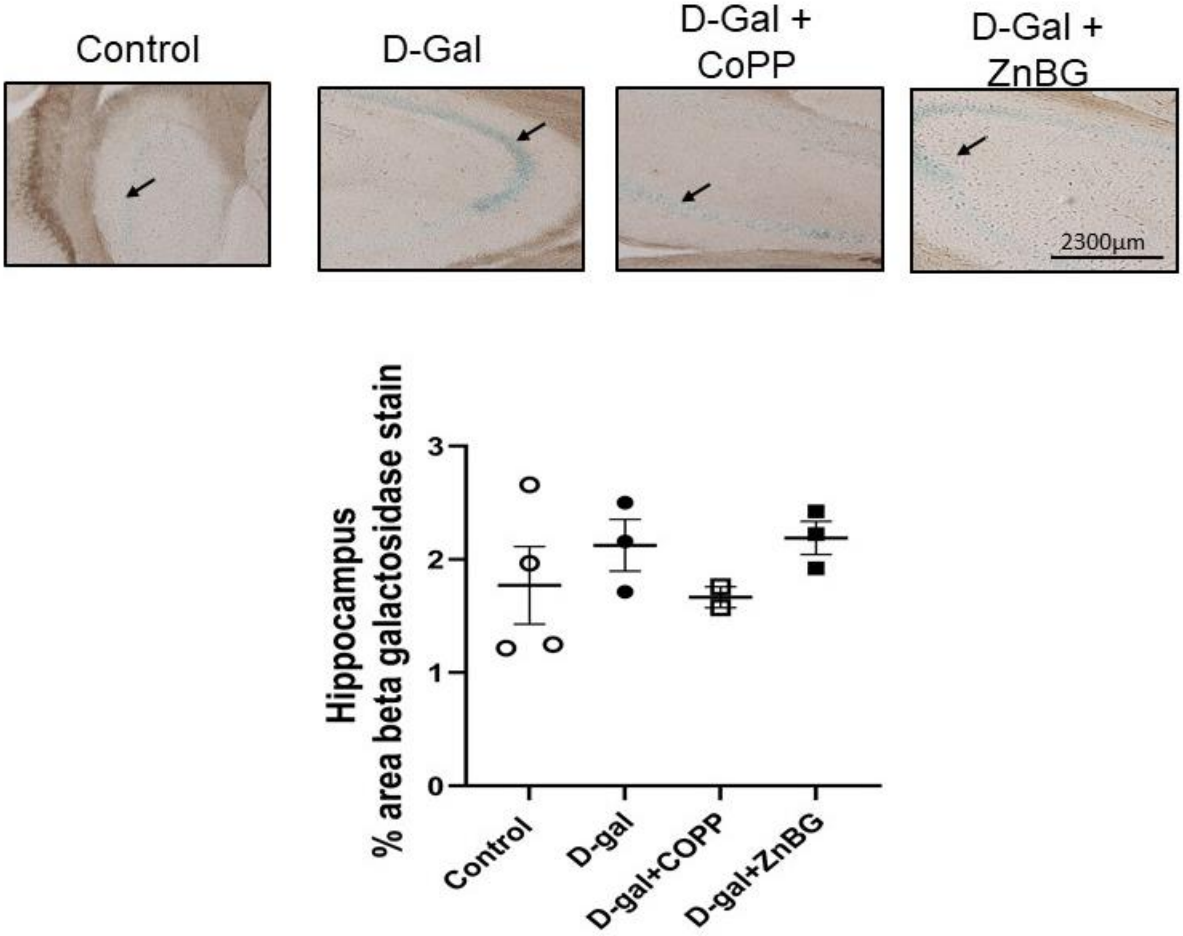

Figure S4. Assessment of hippocampal senescence in male mice. β-galactosidase stain of hippocampal brain sections of male mice. Magnified with x10 lens, field size 2300µm. Statistical analyses were performed using a one-way ANOVA with LSD post hoc test for multiple comparisons. Arrows highlight areas of staining.

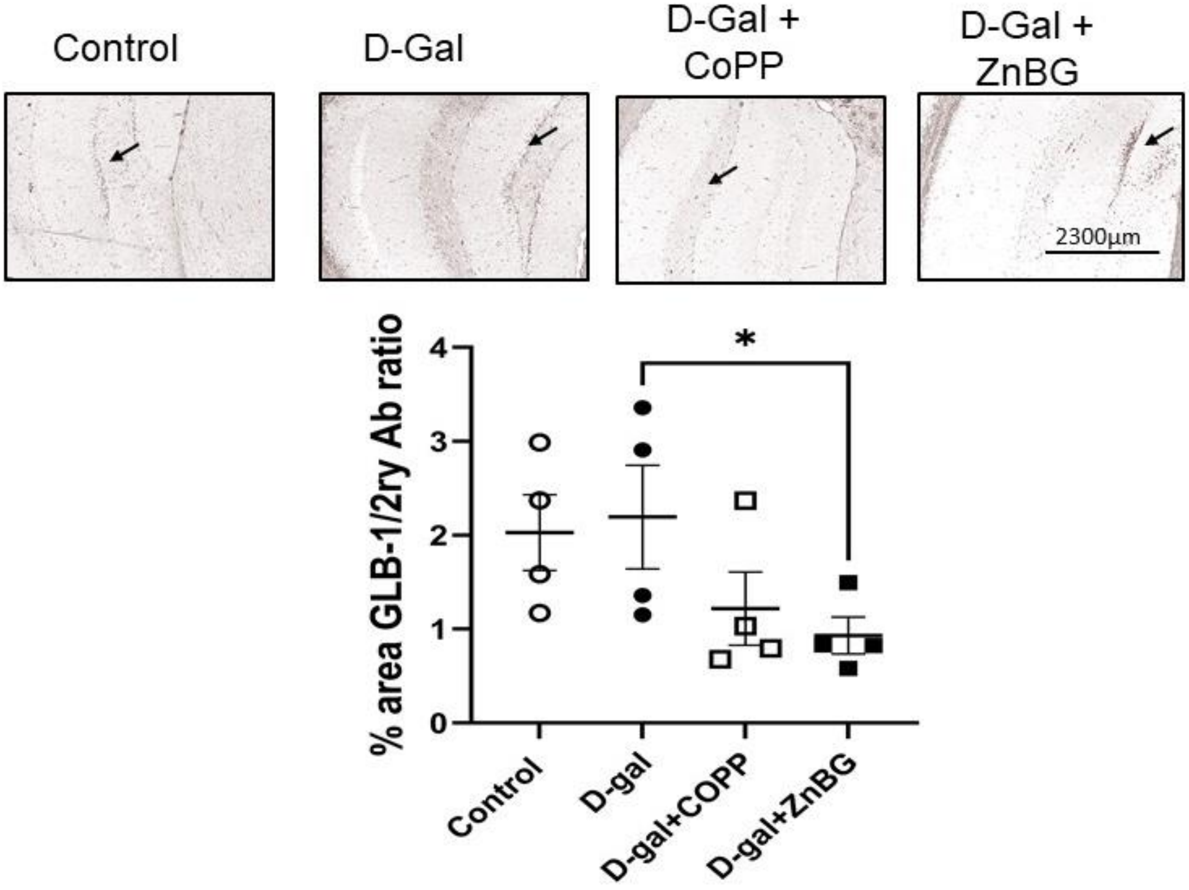

Figure S5. Assessment of hippocampal senescence in female mice. Immunohistochemistry staining of hippocampal sections of female mice with GLB-1 antibodies. Magnified with x10 lens, field size 2300μm. * P <0.05. Statistical analyses were performed using a one-way ANOVA with LSD post hoc test for multiple comparisons. Arrows highlight areas of staining.

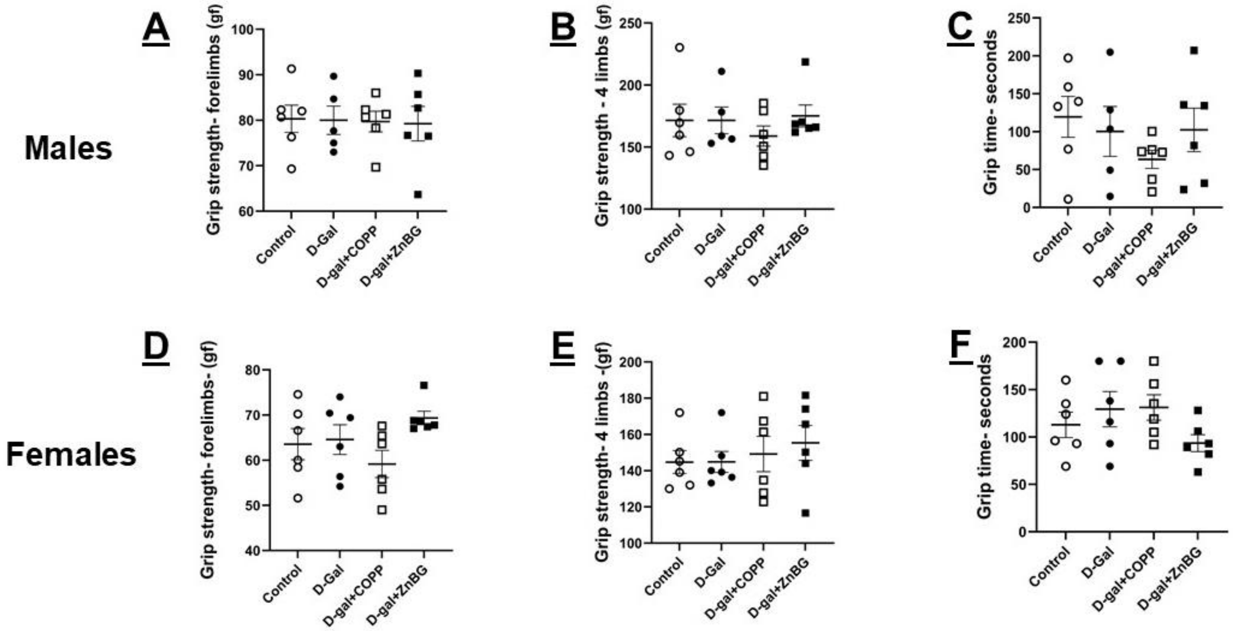

Figure S6. Assessment of grip strength in chronic d-gal-treated mice. Quantitative analysis of (**A**) male forelimbs grip strength, (**B**) male fore-and hindlimbs grip strength, (**C**) male mice grip time in 4-limb inverted hanging test, (**D**) female forelimbs grip strength, (**E**) female fore-and hindlimbs grip strength, (**F**) female mice grip time in 4-limb inverted hanging test. Values are expressed as mean ± SEM, *n*= 6. CON; control group, D-gal; d-galactose model group. Statistical analyses were performed using a one-way ANOVA with LSD post hoc test for multiple comparisons.

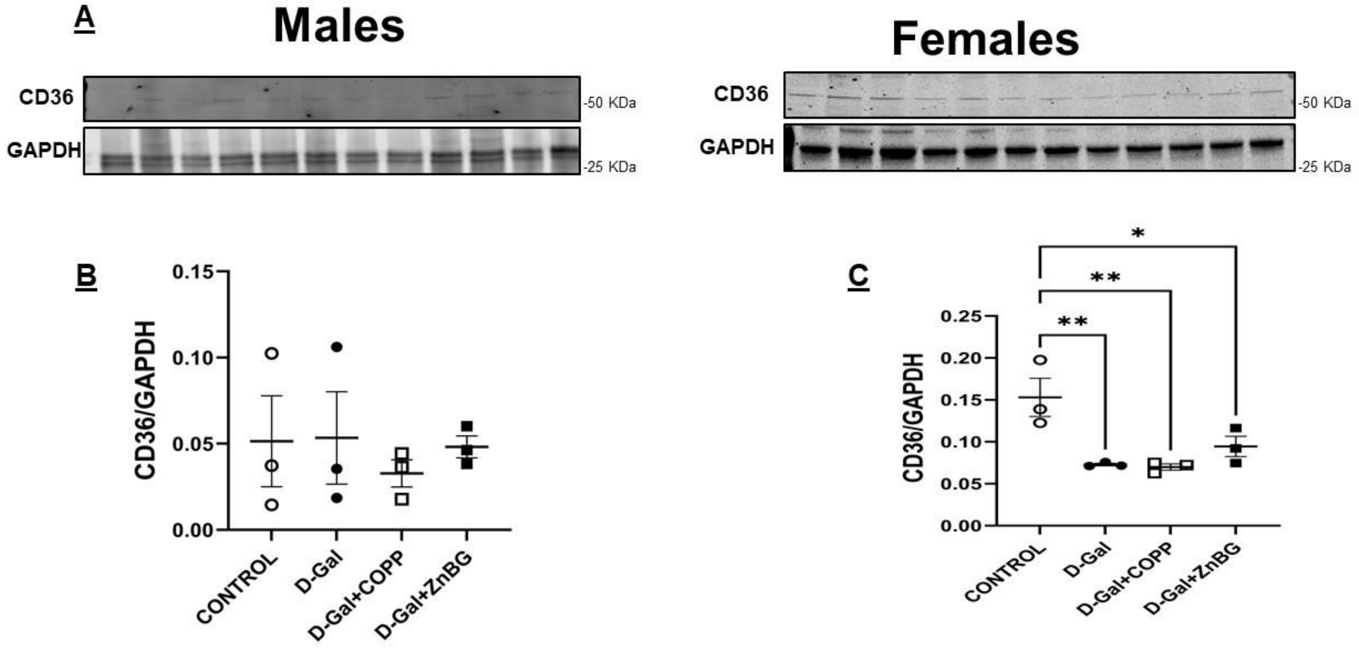

Figure S7. Assessment of CD36 protein levels in the hindlimb muscles of d-gal-treated mice. (**A**) Representative Western blots from male and female mice. (**B**) Quantitation of CD36 in the muscle from male mice. (**C**) Quantitation of CD36 in the muscle from female mice. Values are expressed as mean ± SEM, * P <0.05, ** P <0.01, *n*= 3, GAPDH; Glyceraldehyde-3-Phosphate Dehydrogenase, Statistical analyses were performed using a one-way ANOVA with LSD post hoc test for multiple comparisons.
